# Correction: Artificial intelligence tool for the study of COVID-19 microdroplet spread across the human diameter and airborne space

**DOI:** 10.1371/journal.pone.0315239

**Published:** 2024-12-05

**Authors:** The PLOS ONE Staff

There are errors in the author affiliations. The correct affiliations are as follows:

Hesham H. Alsaadi^1^, Monther Aldwairi^1,2^, Faten Yasin^3^, Sandra C. P. Cachinho^4^, Abdullah Hussein^5^

**1** College of Technological Innovation, Zayed University, Abu Dhabi, UAE, **2** Department of Network Engineering and Security, Jordan University of Science and Technology, Irbid, Jordan, **3** Department of Molecular and Clinical Cancer Medicine, University of Liverpool, Liverpool, United Kingdom, **4** Cell Sorting and Isolation Facility, Research Technology Building, University of Liverpool, Liverpool, United Kingdom, **5** Rochester Institute of Technology, Dubai, UAE

The publisher apologizes for the error.
