# Supplementary material for: Artificial intelligence tool for the study of COVID-19 microdroplet spread across the human diameter and airborne space
Source: PLoS One. 2023 Jul 19;18(7):e0269905. doi: 10.1371/journal.pone.0269905 (PMC10355432; doi:10.1371/journal.pone.0269905)
Supplement: Attachment — Submitted filename: Response to Reviewers.pdf [file pone.0269905.s001.pdf]

Summary of Revision

Dear reviewers,

We deeply appreciate your valuable and constructive comments and we thank you for the review, because it ultimately helped improve the manuscript. We worked diligently and made significant efforts to address and to carry out all essential revisions suggested by you. We are confident that this revised manuscript reflects most of the reviewers' comments.
We hope this revised version is satisfactory and will lead to a positive decision. We detail our responses below.

Editorial Comments

We thank the editor for his valuable comments.

1. Please ensure that your manuscript meets PLOS ONE's style requirements, including those for file naming. The PLOS ONE style templates can be found at
https://journals.plos.org/plosone/s/file?id=wjVg/PLOSOne_formatting_sample_main_body.pdf and
https://journals.plos.org/plosone/s/file?id=ba62/PLOSOne_formatting_sample_title_authors_affiliations.pdf.

We are using PLOS ONE LaTeX templates and adhering to the style requirements referenced above.
Action and changes to the paper
Authors emails have been removed from authors list and formatting fixed

2. Thank you for stating the following financial disclosure:
MA
Research Incentive Fund Grant #R20089
Zayed University Research Office
www.zu.ac.ae
NO.

Please state what role the funders took in the study. If the funders had no role, please state: "The funders had no role in study design, data collection and analysis, decision to publish, or preparation of the manuscript."
If this statement is not correct you must amend it as needed.
Please include this amended Role of Funder statement in your cover letter; we will change the online submission form on your behalf.

The funders did not play any role and we added the statement to the cover letter.
Please update the submission from your side.

3. Thank you for stating the following in the Acknowledgments Section of your manuscript:
This work was supported by Zayed University Research Office, Research Incentive Fund Grant #R20089
We note that you have provided funding information that is not currently declared in your Funding Statement. However, funding information should not appear in the Acknowledgments section or other areas of your manuscript. We will only publish funding information present in the Funding Statement section of the online submission form.
Please remove any funding-related text from the manuscript and let us know how you would like to update your Funding Statement. Currently, your Funding Statement reads as follows:
MA
Research Incentive Fund Grant #R20089
Zayed University Research Office
www.zu.ac.ae
NO
Please include your amended statements within your cover letter; we will change the online submission form on your behalf.

This is the only funding information, we removed it from acknowledgement section.
Action and changes to the paper

Removed from acknowledgement

4. We note that you have stated that you will provide repository information for your data at acceptance. Should your manuscript be accepted for publication, we will hold it until you provide the relevant accession numbers or DOIs necessary to access your data. If you wish to make changes to your Data Availability statement, please describe these changes in your cover letter and we will update your Data Availability statement to reflect the information you provide.

I believe what we meant was the code for COVNET45 tools. All the data used is in the public domain!
Please change our Data Availability statement accordingly.

5. Please upload a copy of Figure ??, to which you refer in your text on pages 5 and 12. If the figure is no longer to be included as part of the submission please remove all reference to it within the text.

The reference to Figure 1 was fixed, and consequently the references to the figure in pages 5 and 12 were fixed.

Action and changes to the paper

Uncommented the Figure in LaTeX and commended the figure upload

6. Please ensure that you refer to Figure 11 in your text as, if accepted, production will need this reference to link the reader to the figure.

When we fixed the reference to Figure 1, all Figure numbers have been changed.
Figure 11 is now referenced on page 11.

7. We note that Figures 2, 4, 5, 6, 7, 8 and 9 in your submission contain copyrighted images. All PLOS content is published under the Creative Commons Attribution License (CC BY 4.0), which means that the manuscript, images, and Supporting Information files will be freely available online, and any third party is permitted to access, download, copy, distribute, and use these materials in any way, even commercially, with proper attribution. For more information, see our copyright guidelines: http://journals.plos.org/plosone/s/licenses-and-copyright.

Please note that all figure numbers have been changed according to the citations.
All of the figures are available under open license as follows (Except one figure that we obtained the permission from the owner) (used in Figs 5 and 6).

Fig1.(B-I) and Fig3.(a)

- IMAGE LINK: https://phil.cdc.gov//PHIL_Images/23591/23591_lores.jpg
- IMAGE WEBSITE: https://phil.cdc.gov/Details.aspx?pid=23591
- Copyright: Attribution 4.0 International (CC BY 4.0)
- Pls check the owner clarification Attached and I quote "Thank you for contacting us with your request to use material published in Preventing Chronic Disease (PCD). PCD is a government publication in the public domain. No copyright exists for published material and all content may be used, shared, and distributed as you wish; provided that proper credit is given to the author(s) of the article(s) and Preventing Chronic Disease. We recommend using the suggested citation provided in the article itself. Please let us know if you have any further questions or concerns"

Fig.2 is ours.
Fig.4 is ours.
Fig.5 (A) (previously Fig.6)

- IMAGE LINK: https://covid19.med.hku.hk/-/media/K2/Downloadables/The-Virus/nCoV2019-Square.ashx?la=en
- IMAGE WEBSITE: https://covid19.med.hku.hk/en/downloads/virus
- Copyright: We obtained the permission of the owner (JOHN NICHOLLS). (email attached)

Figs 6 (previously Fig 10)

- Copyright: We obtained the permission of the owner (JOHN NICHOLLS). (email attached)

Fig.7 (A) (previously Fig.8)

- Viola, I. M., Peterson, B., Pisetta, G., Pavar, G., Akhtar, H., Menoloascina, F., ... & Mehendale, F. V. (2021). Face coverings, aerosol dispersion and mitigation of virus transmission risk. IEEE Open Journal of Engineering in Medicine and Biology, 2, 26-35.
- PAPER: https://ieeexplore.ieee.org/abstract/document/9329130
- IMAGE: https://ieeexplore.ieee.org/mediastore_new/IEEE/content/media/8782705/9299466/9329130/viola3-3053215-large.gif

COPYRIGHT: Attribution 4.0 International (CC BY 4.0)

Fig.8 (A) (previously Fig 5)

Kowal, M., Żejmo, M., Skobel, M., Korbicz, J., & Monczak, R. (2020). Cell nuclei segmentation in cytological images using convolutional neural network and seeded watershed algorithm. Journal of Digital Imaging, 33(1), 231-242.

PAPER: https://link.springer.com/article/10.1007/s10278-019-00200-8

FIGURE LINK: https://link.springer.com/article/10.1007/s10278-019-00200-8/figures/7

Copyright: Attribution 4.0 International (CC BY 4.0)

Fig.8 (C) (previously Fig 5)

Cai, Y., Huang, T., Chen, L., Gao, S., & Zhang, N. (2014). Novel computational methods and tools in biomedicine and biopharmacy. Computational and Mathematical Methods in Medicine, 2014.

PAPER: https://www.hindawi.com/journals/cmmm/2014/401201/

FIGURE LINK: https://www.hindawi.com/journals/cmmm/2014/401201/fig1/

copyright: Copyright © 2014 Yu Guo et al. This is an open access article distributed under the Creative Commons Attribution License, which permits unrestricted use, distribution, and reproduction in any medium, provided the original work is properly cited.

Fig.9 (A,B) (previously Fig.7)

Stadnytskyi, V., Bax, C. E., Bax, A., & Anfinrud, P. (2020). The airborne lifetime of small speech droplets and their potential importance in SARS-CoV-2 transmission. Proceedings of the National Academy of Sciences, 117(22), 11875-11877.

PAPER: https://www.pnas.org/doi/full/10.1073/pnas.2006874117

FIGURE LINK: https://www.pnas.org/cms/10.1073/pnas.2006874117/asset/03a58387-760c-4c37-9d6a-bb2fef5bc447/assets/graphic/pnas.2006874117fig01.jpeg

Copyright: Copyright © 2020 the Author(s). Published by PNAS. This open access article is distributed under Creative Commons Attribution License 4.0 (CC BY).

Fig.10 (A,B,C,D) (previously Fig.9)

Pretorius, E., Venter, C., Laubscher, G. J., Lourens, P. J., Steenkamp, J., & Kell, D. B. (2020). Prevalence of amyloid blood clots in COVID-19 plasma.

PAPER: https://www.medrxiv.org/content/10.1101/2020.07.28.20163543v1.full-text

FIGURE LINK: https://www.medrxiv.org/content/medrxiv/early/2020/07/29/2020.07.28.20163543/F4.large.jpg

COPYRIGHT: Attribution 4.0 International (CC BY 4.0)

Action and changes to the paper

The figure captions of the copyrighted figures was modified to show the following text.

Reprinted from [20] under a CC BY license, with permission from LKS Faculty of Medicine, and Electron Microscopy Unit, The University of Hong Kong, original copyright 2019.

Additional Editor Comments:

1. There is no new innovations with proposed framework, Whatever stated is commonly available with all the COVID-19 microdroplet spread.

The article proposes and evaluates a complete framework and software that uses five techniques as well as machine learning for image optimization, object detection, visualization and measurement. This definitely sets it apart from the other image analysis work in the literature as discussed in the related work section.

The main aim is to develop a novel image optimization technique for measuring the distance of microdroplets spread and transmissions in airborne space.

Action and changes to the paper

The contributions were modified to highlight the unique features of this framework as well highlighting the use of machine learning as follows. Pls check the response to reviewers 1 below for more details.

OpenCV includes is an open sources library with numerous pre-trained machine learning classifiers for computer vision problems such as object detection. OpenCV hides the complexity of machine learning such as feature selection, classifier selection and training, and other mathematical complexity. It is used as for detection in COVNET45.

2. How the super speech emitters having specimens and feature fusion model proposed with classifiers.

Our work is not concerned directly with advancing Covid-19 and transmission mechanisms from the medical and biological perspectives. However, we are focused on images processing models that may assist and enable physicians and other medical partitioners diagnose Covid-19 and analyze the spread patterns.

3. The quality of language satisfies. But the manuscript needs to be edited for grammar and syntax.
Noted.
Action and changes to the paper
The manuscript has been thoroughly proofread (see marked version)

4. Spell out each acronym the first time used in the body of the paper. Spell out acronyms in the Abstract only if used there.
Noted.
Action and changes to the paper
Revised and defined all acronyms.

5. Make sure that the Conclusion briefly summarizes the results of the paper it should not repeat phrases from the Introduction.
Done.
Action and changes to the paper
Conclusions section has been revised.

Reviewer #1:

1. The authors are required to make major revisions to the manuscript in terms of clarity of presentation. In the present form, the methodology of the COVNET45, its performance, and the interpretation of the results appear unclear.
We proofread the manuscript and edited many paragraphs from the methodology, performance and results interpretation. We feel that the technical description of the framework, while challenging to none computer scientists to follow, it is still clear to those with expertise in the field of raster image processing.

Action
The manuscript has been thoroughly proofread.

2. Also, it is not clear how this would benefit the scientific community and the governments to control the COVID-19 pandemic. Moreover, does this application has benefits in other diseases, etc. is not explored by the authors. An in-depth and professional language editing may be essential.
COVNET45 explores the design aspect of a framework to be applied in the research in medical practices, the selection of the specific algorithms that has been proposed such as SOTA in their relative fields have been embedded in the design aspect of the COVNET45 application framework. We proposed and evaluated a complete framework and software that uses five techniques as well as machine learning for image optimization, object detection, visualization and measurement. COVNET45 used OpenCV with numerous pre-trained machine learning classifiers to perform object detection.

It is true that the technique and tool may be useful in other airborne diseases. However, the selection of COVID-19 was due to the interest of authors to design a framework around it. The tested cases of Covid are selected to be positive in this research to validate the data and can be assisted later to design a different approach of a framework that can be tested for classification. Finally, other diseases can be tested in future.

Action and changes to the paper
The contributions have been rewritten as follows.

In this paper, we propose an intelligent technique to visualize, detect, and measure the distance of the spread of microdroplet transmissions in airborne space in a real-world setting. The article proposes and evaluates a complete framework and software that uses five techniques as well as machine learning for image optimization, object detection, visualization and measurement. The main aim is to develop a novel image optimization technique for measuring the distance of microdroplets spread and transmissions in airborne space.

The main contributions of this work are:

1. Generate high accuracy measurement results by optimizing the collected images and their characterization.
2. Combine the strong capabilities of accurately measuring spread with the use of different scale systems: nominal, ordinal, interval and ratio.
3. Provide a generic approach that is not limited to small scale microdroplets transmissions, but also able to measure the diameter of spread in any space using 2D graphical images with the capabilities of analyzing, optimizing and detecting various semantic marks.
4. Provide a versatile tool capable of analyzing different images formats, which may enable researcher to authenticate previous research results and compare to related work
5. Latest AES-256 encryption is used to protect the hosted images in AWS cloud to ensure added privacy.

Reviewer #2:

1. This article devoted of using the specific software for images and drops measurement which appear in the process sneezing or cough or breathing or talking. These drops might appear not only in COVID-19 infected persons, but in influenza and other respiratory diseases infected persons (not only viral but also bacterial infections). Therefore - the title of the article with COVID-19 not appropriate. Perhaps this new program software may be use for different aims, but it not allowed to differentiation of drops of one infectious diseases from other. My proposition for authors - to corrected this article.

We thank the reviewer for this observation. It is true that the technique and tool are useful for other airborne diseases, however it is the authors position is that the title is appropriate since the images used to study the efficiency of the technique was Covid-19 related.
The authors will continue to study the proposed technique and improve the tool through testing with other airborne diseases' images.

Action and changes to the paper
N/A

2. Besides in the article on the pages 5 and 12 not indicated the Figure's number (Fig.??).
Action
All figures' references have been fixed.
