# Supplementary material for: Artificial intelligence tool for the study of COVID-19 microdroplet spread across the human diameter and airborne space
Source: PLoS One. 2023 Jul 19;18(7):e0269905. doi: 10.1371/journal.pone.0269905 (PMC10355432; doi:10.1371/journal.pone.0269905)
Supplement: Attachment — Submitted filename: Response to Reviewers.pdf [file pone.0269905.s002.pdf]

Summary of Revision

Dear reviewers,

We deeply appreciate your valuable and constructive comments and we thank you for the review, because it ultimately helped improve the manuscript. We worked diligently and made significant efforts to address and to carry out all essential revisions suggested by you. We are confident that this revised manuscript reflects most of the reviewers' comments.
We hope this revised version is satisfactory and will lead to a positive decision. We detail our responses below.

Reviewer #1:

1. All comments have been addressed

Thank you.

2. Thanks for making the necessary changes. Unfortunately, the non-computer field scientists would still feel difficult to understand this paper

Action

This is a technical paper with the expected audience made of technology scientists working on building image analysis tools to assist those in the medical fields. We do not expect Physicians to totally understand everything in the paper.

Action and changes to the paper

Nonetheless, the manuscript has been thoroughly proofread (see the marked version).

Reviewer #3:

1. Reviewer #3: -The paper should be interesting ;;;
-it is a good idea to add more photos of measurements, sensors + arrows/labels what is what (if any);;;
-figures should have high quality. ;;;;;

We thank the reviewer for this observation. Due to space and production quality, we cannot include all images, this is a continuing work, therefore we have published the tool and more images publicly on GitHub as follows
https://github.com/HeshamAlsaadi/COVNET45/tree/main/Research%20Images
Original Sources Link and README file
https://github.com/HeshamAlsaadi/COVNET45/blob/main/README.md

More images and diagrams will be added as soon as there are produced.

Action and changes to the paper

N/A

2. -What is the result of the analysis?;;
-what will society have from the paper?;;
-please add photos of the application of the proposed research, 2-3 photos ;;;

The tool will support scientists from all variety of fields, mainly IT developer who device tools for medical image analysis. COVNET45will allow them to harness the power and the use of AI technologies using several automatic intelligent techniques to process, detect and measure any giving object(s) and event(s) in raster image files highlighted in this paper with a discussion of microdroplet transmissions in airborne space. For example, biologist uses microscopic to study biological data to analyze cellular structure, organism, and characteristics. Understanding different cell characteristic under different conditions like germs or viruses, and effect of cell proliferation to examine the other conditions in-depth using AI algorithmic software's. COVNET45 can examine anatomical cell and measure characteristics like capturing, tracking cells like microscopic studies a common tool as ImageJ.

Action and changes to the paper

Please note that we have highlighted the results and contributions of this work in the abstract, introduction and new edited conclusions

3. -text should be formatted;;;;
-labels of figures should be bigger;;;;

We thank the reviewer for his comments, however, please note that the text is formatted as per the journal template. The same applies to figures captions, those are dictated by the journal style sheets.

Action and changes to the paper

N/A

4. -Is there a possibility to use the proposed research for other topics:

"Role of Hybrid Deep Neural Networks (HDNNs), Computed Tomography, and Chest X-rays for the Detection of COVID-19",;;;
"A Novel Method for COVID-19 Diagnosis Using Artificial Intelligence in Chest X-ray Images",;;;
We thank the reviewer for this comment. Yes, COVET45 may be used as a measurements tool to accurately examine anatomical cells and measure characteristics like capturing, tracking cells for microscopic studies. Also, it can examine analyze distance and scale based in world scale system.
As for the X-ray images, COVNET45 is currently hosted in AWS and can be integrated with the AMAZON AI machine-learning platform. It is possible to extend the implementation of the block level representation of COVNET45 using hybrid deep neural network (HDNNs). Moreover, this can be implemented as a feature classification model in the tool if the model is trained with accurate and large dataset.

In short, the model presented in the paper can be implemented; the trained model will allow us to detect any anomalies with precision and accuracy. However, this out of the scope of this publication for the time being.

Action and changes to the paper
N/A

5. -references should be from the web of science 2020-2022 (50% of all references, 30 references at least);;;

We thank the reviewer for his effort to ensure proper and high-quality sources are cited, we have developed and authored the work during 2020. That is why majority of the citation 15 out of 24 are from 2020 when the pandemic was at its peak. However, the nature of scholarly publication delays in submission, review and other factors may lead to citation looking old.

Pls note that the reason behind this work was the limited reliable research that study microdroplets spread and transmissions. We tried our best to cite newly published work during 2021 and 2022 time focusing on web of science and other reputable venues.
Action
The following papers have been discussed and cited.

14. HeshamAlsaadi, Aldwairi M. COVNET45: Artificial intelligence tool for the study of COVID-19 microdroplet spread across the human diameter and airborne spac; 2022. Available from: https://github.com/HeshamAlsaadi/COVNET45/blob/main/README.md.
15. HeshamAlsaadi, Aldwairi M. COVNET45 Images; 2022. Available from: https: //github.com/HeshamAlsaadi/COVNET45/tree/main/Research%20Images.
22. Yamamoto M, Kawamura A, ichi Tanabe S, Hori S. Predicting the infection probability distribution of airborne and droplet transmissions. Indoor and Built Environment. 0;0(0):1420326X221084869. doi:10.1177/1420326X221084869.
23. de Oliveira PM, Mesquita LCC, Gkantonas S, Giusti A, Mastorakos E. Evolution of spray and aerosol from respiratory releases: theoretical estimates for insight on viral transmission. Proceedings of the Royal Society A: Mathematical, Physical and Engineering Sciences. 2021;477(2245):20200584. doi:10.1098/rspa.2020.0584.
24. Rowe BR, Canosa A, Meslem A, Rowe F. Increased airborne transmission of COVID-19 with new variants, implications for health policies. Building and Environment. 2022;219:109132. doi:https://doi.org/10.1016/j.buildenv.2022.109132.
25. Niedrite L, Arnicans G, Solodovnikova D. Visualization of Indoor Sensor Data to Reduce the Risk of Covid-19 Infection. In: Doctoral Consortium/Forum@DB&IS; 2022.

6. -please compare advantages/disadvantages of other approaches;;;
We thank the reviewer for his useful comments, and we include below several paragraphs that deal with the advantages and disadvantages of the proposed methods in the literature.

Action
New discussion added to related work

The following paragraphs deals with the comparison to related work
Yamamoto et al. proposed a probabilistic mathematical model for the distribution of airborne and droplets to predict Covid-19 infection [22]. For the parameters needed they relied on known estimation methods, however no real measurement or images processing was performed. It is true they applied this prediction model in a real office where they measured CO_2 concentrations and the occupant positions, however there was no image analysis or measurement to study the spread of particles. Similarly, Oliveira et al. [23] presented a theoretical mathematical estimate of virus transmission to provide social distancing and ventilation measures, however none of that is comparable to actual microdroplet measurements capabilities of COVNET45.
More studies have dealt with the issue of airborne contamination paths, counting of pathogens, quantum concentration, carbon dioxide concentration and other measurements to give recommendations about ventilation and indoor capacity [24]. Additionally, there is a wealth of work in the area of Indoor air quality (IAQ) to use sensors and visualization in preventing indoor spread of SARS-CoV2 [25]. We believe those studies could benefit from the measurement capabilities of droplet transmission provided by COVNET45.

Existing discussion dealing with advantages and disadvantages of the proposed methods in the literature.

The research studies, mentioned earlier, adopted different techniques for scaling and measuring microdroplets infection transmissions either by simulating graphical representation of microscopic images or by visualizing the spread using Computational Fluid Dynamics (CFD) examination. Many of the discussed studies lack the evidentiary tools to validate their droplets measurement results, this makes it more complicated to authenticate the results in every research investigating the virus droplets spread through patient cough or sneeze. Some studies also supported only specific data formats

(CT scans, micrographic/microscopic images, CFD images, video graphics), and even when handling multiple formats, they cannot compare or validate results because of the restrictions presented by the investigation technology used. The fact that microdroplets patterns are ideally identifiable and recognizable makes it challenging to measure the precise size, even when using automated scaling systems implemented within the technology. Finally, extensive types of scales' formats for accurately measuring microdroplets or any microscopic details within the hierarchy is challenging, which leaves the door open to investigate more optimized tools to explore and authenticate studies about the infectious disease.

-Conclusion: point out what have you done;;;;

We highlighted the paragraphs below in the conclusions that explain what we have done

Biologist uses microscopic images to study biological data to analyze cellular structure and organism characteristics. Artificial Intelligence algorithmic may assist in understanding different cell characteristic under different conditions such as germ, viruses and effect of cell proliferation.

This paper presented an automatic measurement detection tool, dubbed "COVNET45". The tool provides five automatic intelligent techniques (SEGBON, EDGE, CED, DITHER and OPTIZ) to process, detect and measure any given objects and events in raster image files. COVNET45 will allow scientists to automatically optimize raster images, such as micrograph images of microscopic images, and perform precise measurements observation to any given object or particles. In addition, COVNET45 can examine anatomical cell and measure characteristics like capturing and tracking cells in microscopic studies.
